# Protective Effect of Hydroalcoholic Extract of *Stachys pilifera* on Oxidant-Antioxidant Status in Renal Ischemia/Reperfusion Injuries in Male Rats

**DOI:** 10.1155/2021/6646963

**Published:** 2021-01-29

**Authors:** Zahra Moslemi, Izadpanah Gheitasi, Amir Hossein Doustimotlagh

**Affiliations:** ^1^Student Research Committee, Yasuj University of Medical Sciences, Yasuj, Iran; ^2^Medicinal Plants Research Center, Yasuj University of Medical Sciences, Yasuj, Iran; ^3^Department of Clinical Biochemistry, Faculty of Medicine, Yasuj University of Medical Sciences, Yasuj, Iran

## Abstract

**Background:**

Renal ischemia-reperfusion (I/R) has a pivotal role in the progression of acute renal failure. Reactive oxygen species are considered the major constituents involved in the biochemical and pathophysiological changes that were shown during kidney I/R. The purpose of this study was to examine the renoprotective effects of *Stachys pilifera* ethanolic extract on oxidant-antioxidant status in renal I/R-injuries in male rats. *Material and methods*. Twenty-one male Wistar rats were arbitrarily distributed into 3 groups: sham control (SC), I/R, and I/R + *Stachys pilifera* ethanolic extract (500 mg/kg). The artery and vein of the right kidney were completely blocked, and the right kidney was completely removed in all groups. Then, the left kidney artery was blocked with suture thread for 30 minutes in only I/R and I/R + *SP* extract groups. Kidney function indices, oxidative stress markers, and hematoxylin and eosin staining were investigated in the plasma and kidney tissues.

**Results:**

It was shown that the urine Na and K, fractional excretion of Na and K, and protein carbonyl content markedly increased in the merely I/R group as compared to SC rats, while the administration of *SP* extract markedly reduced these indices (*P* < 0.05). Also, glomerular filtration rate and total thiol meaningfully reduced in the I/R rats in contrast to the SC group, while the treatment with *SP* extract markedly augmented these indices (*P* < 0.05). However, in agreement with renal function tests, *SP* extract had no significant effects on histopathological examinations.

**Conclusion:**

It seems that *SP* extract employs renoprotective effects on renal damage induced by I/R, possibly by improving of oxidant-antioxidant status in favor of the antioxidant system.

## 1. Introduction

Acute kidney injury or acute renal failure (ARF) is an immediate loss of renal function that is connected with high mortality [1]. Ischemia-reperfusion (I/R) is a usual complication during partial nephrectomy, kidney transplantation, hydronephrosis, and cardiopulmonary bypass, which lead to kidney dysfunction and damage [2]. The most important cause of ARF is kidney I/R damage. There is no specific treatment for ARF patients [3]. Heart failure, dialysis, and sepsis are main causes of death in ARF patients in developed countries [4]. Reactive oxygen species (ROS), purine metabolites, vasodilators, and activated neutrophils involved in the pathogenesis of I/R damage [5, 6]. It has been stated that diverse cellular pathways such as oxidative stress and inflammation play a pivotal role in the acute renal ischemia. Actually, extreme formation of ROS leads to production of cytotoxic metabolites, which can induce irreversible disorders such as lipid peroxidation and DNA damage [7]. These changes were in agreement with the worsening of kidney function, signifying that renal I/R induced kidney ROS formation, reduced the capacity of cells to remove ROS, augmented endogenous antioxidant depletion, and deteriorated renal injury [8, 9].

In previous studies, the protective impact of *Malva sylvestris* L. [10], *Origanum majorana* L. [11], and *Salvia miltiorrhiza* [12] have been explored on the I/R-induced damage. *Stachys pilifera Benth* (*SP*) is one of genera in the family of Lamiaceae, and the genus *Stachys* is distributed in the tropical and subtropical regions [13, 14]. *Stachys* has about 300 species, and those thirty-four have been described in the flora of Iran of which 13 species are native [13, 15]. It has antibacterial [16], antioxidant [17], anti-inflammatory [18], antitumor and antidiabetic [19], hepatoprotective [14], immune protection [20], antihepatitis [21], and disinfectant [13] properties. The aerial parts of this plant are used in traditional Iranian medicine as herbal tea to treat various disorders such as asthma, infection, and rheumatoid arthritis [14, 15]. Since chemical drugs have some side effects and obstacles, trend in the direction of traditional medicine and medicinal plants is rising in the world. *Stachys pilifera* has inordinate antioxidant properties; no studies have been done on the effect of it on I/R-induced renal failure. Then, the purpose of the present study was to evaluate the effects of *SP* ethanolic extract on renal damages induced by I/R in male Wistar rats.

## 2. Material and Methods

### 2.1. Preparation of *Stachys pilifera* Extract

The aerial parts of *SP* were collected by an expert in the spring of 2019 from Yasuj mountain (Yasuj, Iran), and it was recognized by the botanist (herbarium no. 1897). The aerial sections of the plant (200 g) were dried, crushed, and soaked in 70% (v/v) ethanolic solution at 25°C for 48 h under shaking station. The obtained extract was dried in an incubator at 50°C and maintained in a freezer at −20°C until starting the study [22].

### 2.2. Experimental Design

Twenty-one male Wistar rats (300 ± 50 g) were obtained from the Yasuj University of Medical Sciences (Yasuj, Iran) and were adopted for one week with access to water and standard diet at 12 h light and dark cycle. All processes were acknowledged by the Ethics Committee of Yasuj University of Medical Sciences (ethical code IR.YUMS.REC.1399.171).

The rats were arbitrarily distributed into three groups as follows: sham control (SC), I/R (positive control), and I/R + 500 mg/kg of *SP* ethanolic extract. Sham control and I/R groups received olive oil 72, 48, and 24 h before the study and immediately after surgery by intraperitoneally [23]. Also, the I/R + *SP* extract group received 500 mg/kg of *SP* ethanolic extract 72, 48, and 24 h before the study and instantly after surgery by orally [24].

The animals were anesthetized by injection of pentobarbital sodium (60 mg/kg). The abdomen was shaved with a safety razor and sterilized with povidone iodine solution. The renal arteries in both kidneys were carefully cleaned and separated using a microscope. The artery and vein of the right kidney were completely blocked using suture thread, and the right kidney was completely separated. The left renal artery was blocked with suture thread for 30 minutes, and after this time, renal blood flow was restored [25]. In SC rats, all surgical procedures were performed, but the left renal artery was not blocked. After regaining consciousness, the animals were transferred to their shelves.

After 24 h of reperfusion, the animals were reanesthetized again with pentobarbital sodium. After shaving the throat, a chip was placed in the trachea for oxygenation. The body temperature was controlled at 37 ± 1°C and connected to a thermistor. After shaving, the left femoral artery was cannulated to record blood pressure and arterial blood sampling, as well as femoral vein cannulation was performed to inject anesthetic and normal saline. To permanently record the animal's blood pressure, the femoral artery was connected to the PowerLab blood pressure monitoring system. Bladder cannulation was done to collect animal urine, and urine samples were collected in preweighed containers. After these measures, in order to relieve the stress of surgery and cannulation, the animals were rested for one hour and then urine sample was collected for 2 h. Arterial blood samples were prepared from femoral cannula, centrifuged, and the plasma was stored in the freezer to measure the levels of sodium (Na), potassium (K), urea, creatinine (Cr), etc. At the end of the study, the left kidney was removed and the capsule part was cleaned and placed on dry ice, weighed, and divided into two parts. The first part was placed in cryotube for determination of oxidative stress markers, and the second part was kept in 10% formalin for histological studies [26, 27].

### 2.3. Biochemical Analysis

Plasma and urine samples were assayed for urea and Cr using commercially available kits (Pars Azmoon, Iran). Na and K were measured by ion selective electrode procedure. The urine flow rate per gram of each kidney weight (V° ml/min gKW) was calculated, as well as urine volume from the left kidney was determined gravimetrically. Moreover, Cr clearance as an estimation of glomerular filtration rate (GFR), absolute excretion of K (UKV°) and Na (UNaV°), and fractional excretion of K (FEK) and Na (FEK) were determined by typical formulae.

### 2.4. Oxidative Stress Markers

The plasma levels of malondialdehyde (MDA) were assayed based on the reaction of MDA with thiobarbituric acid. The MDA value was determined using a molar absorption coefficient of 1.56 × 105 M^−1^ cm^−1^ and indicated as *μ*mol/L [28]. Protein carbonyl (PCO) groups were measured using dye-based spectrophotometry based on the reaction with 2, 4-dinitrophenylhydrazine. PCO level was estimated using the molar absorption coefficient of 2.2 × 10^4^ M^−1^ cm^−1^ and was determined as *μ*mol/g tissue [28]. Tissue homogenate and plasma total thiol (T-SH) were determined according to the reaction of 5, 5′-dithiols-(2-nitrobenzoic acid) with thiol groups and creation of 2-nitro-5-thiobenzoic acid. The T-SH content was estimated utilizing the molar absorption coefficient of 13,600 M^−1^ cm^−l^ [29]. The ferric reducing antioxidant power (FRAP) was assayed based on the capacity of plasma or kidney homogenate in restoration of ferric ion (Fe^3+^) to ferrous ion (Fe^2+^) in the presence of tripyridyl-s-triazine reagent [30].

### 2.5. Histological Examinations

For histological analysis, renal tissue specimens were removed and fixed in 10% formalin. After embedding, the kidney tissues were cut into 3–4 *μ*m pieces. The pieces were mounted and stained with hematoxylin and eosin (H&E) staining and observed by a pathologist that who was blinded to the groups.

### 2.6. Statistical Analysis

Data were assessed utilizing one-way ANOVA test following by Tukey's multiple comparison. The data were shown as mean ± SEM. *P* < 0.05 level was considered statistically significant in this study.

## 3. Results

### 3.1. Biochemical Markers

**Figure 1:**
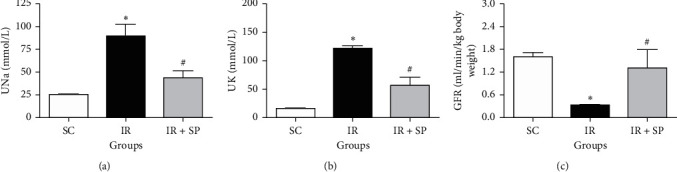
Effect of *SP* extract on UNa (a), UK (b), and GFR (c) following I/R injury. Data are shown as mean ± SEM. ^*∗*^Significant against the SC group (*P* < 0.001); ^#^significant against the IR group. SC: sham control; *SP*: *Stachys pilifera*; I/R: ischemia-reperfusion; UNa: urine Na (mmol/L); UK: urine K (mmol/L); and GFR: glomerular filtration rate (ml/min/kg body weight).

As shown in Figure 1, urine Na and K were obviously enlarged in the I/R untreated group in contrast to SC rats (*P* < 0.05), while the treatment with *SP* ethanolic extract markedly declined these markers (*P* < 0.05). GFR was significantly reduced in I/R untreated rats against the SC group (*P* < 0.05), while treatment with *SP* extract markedly increased this marker.

**Figure 2:**
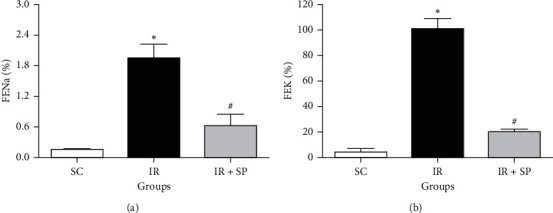
Effect of *SP* extract on FENa (a) and FEK (b) following I/R injury. Data are shown as mean ± SEM. ^*∗*^Significant against the SC group (*P* < 0.05); ^#^significant against the IR rats (*P* < 0.05). SC: sham control; *SP*: *Stachys pilifera*; I/R: ischemia-reperfusion; FENa: fractional excretion of Na (%); and FEK: fractional excretion of K (%).

The fractional excretion of K and Na (FEK and FENa) was markedly enlarged in the I/R group in comparison to SC animals, while treatment with SP extract meaningfully decreased these indices (Figure 2).

The UNaV° and UKV°, as well as plasma urea and Cr, were markedly increased in the I/R rats in contrast to the SC group; however, consumption of *SP* extract slightly reduced UNaV°, UKV°, and Cr levels (Table 1).

### 3.2. Oxidative Stress Markers

**Figure 3:**
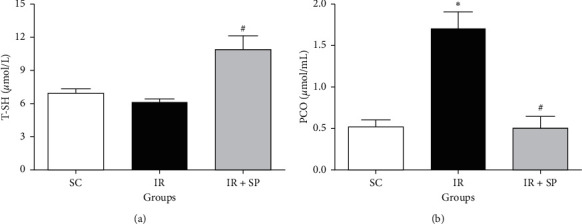
Effect of *SP* extract on T-SH (a) and PCO (b) following I/R injury. Data are shown as mean ± SEM. ^*∗*^Significant against the SC rats (*P* < 0.05); ^#^significant against the IR group (*P* < 0.05). SC: sham control; *SP*: *Stachys pilifera*; I/R: ischemia-reperfusion; T-SH: total thiol; PCO: protein carbonyl.

The plasma T-SH was slightly reduced, and the plasma PCO evidently increased in the merely I/R rats in comparison to SC rats; however, the consumption of *SP* extract significantly abrogated these parameters (*P* < 0.05 (Figure 3).

**Figure 4:**
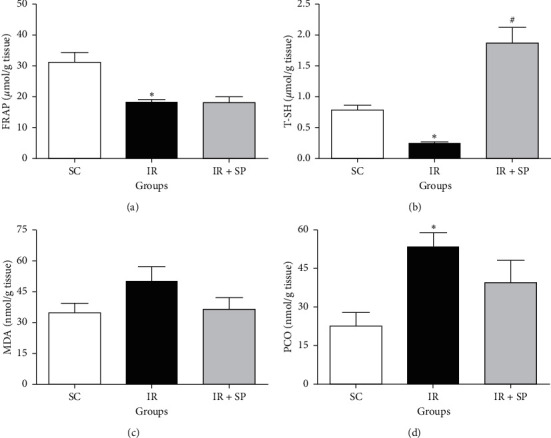
Effect of *SP* extract on FRAP (a), T-SH (b), MDA (c), and PCO (d) following I/R injury. Data are shown as mean ± SEM. ^*∗*^Significant against the SC rats (*P* < 0.05); ^#^significant against the I/R group (*P* < 0.05). SC: sham control; *SP*: *Stachys pilifera*; I/R: ischemia-reperfusion; FRAP: ferric reducing antioxidant power; PCO: protein carbonyl; T-SH: total thiol; MDA: malondialdehyde.

The levels of FRAP and T-SH contents in renal tissue were noticeably reduced in the I/R untreated rats in comparison to the SC group, while the consumption of *SP* ethanolic extract only could significantly augmented T-SH levels as compared to merely I/R animals (*P* < 0.05). The tissue PCO levels were strikingly augmented in I/R rats in contrast to the SC group (*P* < 0.05), whereas treatment of the I/R group with *SP* extract insignificantly reduced it. The MDA level was slightly enlarged in the I/R rats against the SC rats, while the administration of *SP* extract insignificantly reduced it (Figure 4).

### 3.3. Histopathological Studies

**Figure 5:**
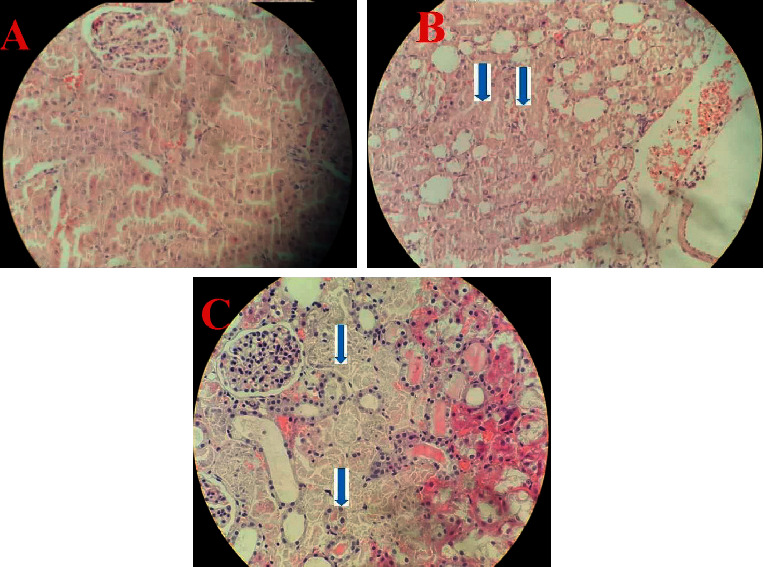
Histopathological findings of kidney tissue stained with hematoxylin and eosin (10x): (a) sham control, (b) I/R, and (c) I/*R* + *SP*; *SP*: *Stachys pilifera*; I/R: ischemia-reperfusion; blue arrow = tubular necrosis.

Renal sections of the SC group showed normal morphology (Figure 5(a)); however, renal tissue of the I/R group demonstrated sever damages such as tubular necrosis, vascular congestion, and white blood cell (WBC) infiltration. However, treatment with *SP* extract had no significant change on these parameters (Figures 5(b) and 5(c).

## 4. Discussion

In this study, we examined the impacts of *SP* ethanolic extract on biochemical, histopathological, and oxidant-antioxidant status during I/R injury. In the current study, renal function was considerably deteriorated in the merely I/R rats by decreasing in the GFR rate, increasing in the urea and Cr levels, as well as worsening of histological changes such as vascular congestion, tubular necrosis, and WBC infiltration. These biochemical and histopathological alterations are in consistent with the previous studies in I/R rats [8, 9] and may be as a result of tubular and vascular changes insulted by oxidative stress [31].

Fractional excretion of K and Na, absolute excretion of K and Na, and urine K and Na were markedly augmented in the I/R untreated rats in contrast to SC rats. In spite of the obviously dropped of GFR in the I/R rats, the significant increment in UKV° and UNaV° was observed in this group; that may be related to defect in the K and Na reabsorption systems especially in the proximal tubule, loss of cell polarity of Na and K ATPase [32], and decrease in ATP level of cell during of I/R [32]. Moreover, increased FENa and FEK as well as decreased GFR in the I/R rats might be due to the severity of proximal tubule damage because proximal tubule cells produce a high level of ROSs following I/R [33]. Treatment with *SP* extract markedly reduced fractional excretion of K and Na, as well as urine K and Na, while it slightly decreased plasma Cr level in comparison to the merely I/R group. Moreover, ethanolic extract of *SP* (500 mg/kg) significantly increased the GFR rate against the only I/R rats. Amendment of the renal function in this study may be as a result of the antioxidant ability of *SP* ethanolic extract, and then trapping of ROSs.

In the current study, renal MDA, as a marker of lipid peroxidation, insignificantly enlarged in the only I/R group against the SC animals. Ahmadvand and Mahdavifard (2019) [34] and Hadj Abdallah et al. (2018) [35] showed that renal MDA levels were elevated in the I/R animals. Also, our results indicated that the kidney MDA level slightly reduced in I/R rats in contrast to I/R + *SP* extract. Our results indicated that plasma PCO, as an early index of protein oxidation, markedly elevated in the I/R group in contrast to SC rats, while *SP* extract (500 mg/kg) significantly reduced it. In agreement with previous studies [11, 35], protein oxidation happens in I/R-induced renal damage. Kang et al. (2006) observed that ROSs play a vital role in the pathogenesis of I/R damage [6]. Furthermore, the genus of *Stachys* contains flavonoids and phenolic compounds [23], which phenolic groups as hydrogen donors were able to react with ROSs and neutralize them [36]. We conclude that treatment with *SP* ethanolic extract could be moderately impede lipid peroxidation and besides absolutely inhibit protein oxidation via inactivating of ROSs.

Thiol groups (including protein thiol groups and glutathione) are sensitive oxidative parameters that defense against ROSs. In consistent with previous study [35], renal T-SH contents were meaningfully reduced in the merely I/R rats in contrast to SC animals, which could be due to antioxidant exhaustion by ROSs. Similar to our preceding studies in acetaminophen-induced hepatotoxicity and cisplatin-induced nephrotoxicity [14, 24], T-SH levels strongly augmented in the I/R + *SP* animals in comparison to only I/R rats. It seems that *SP* extract was able to keep thiol groups storage by neutralizing ROSs or increasing glutathione synthesis.

Oxidative stress has a pivotal role in the progression of renal dysfunction induced by I/R injury. The improvement of oxidant-antioxidant status and renal function in this study indicated that oxidative stress has an essential role in I/R damage, and *SP* ethanolic extract has nephroprotective activity in contrast to I/R-induced renal injury.

## 5. Conclusion

It seems that *SP* extract employs renoprotective effects in I/R injury possibly by improving of some renal function markers, inhibiting of protein oxidation, and repairing of thiol groups storage. The renoprotective activity of *SP* extract may be related to changing of oxidant-antioxidant balance in favor of the antioxidant system. These results recommend that the usage of *SP* ethanolic extract, as a supplemental remedy, may be inhibit renal toxicity induced by I/R injury.

## Figures and Tables

**Table 1:** Effects of *Stachys pilifera* extract on renal function parameters.

Groups	SC	IR	IR + *SP*
KW (gr)	1.07 ± 0.05	1.09 ± 0.05	1.23 ± 0.02
UV (mL)	0.015 ± 0.000	0.012 ± 0.002	0.011 ± 0.001
V° (mL/min gKW)	0.014 ± 0.000	0.010 ± 0.001	0.013 ± 0.002
PNa (mmol/L)	146.35 ± 2.58	147.60 ± 1.53	147.11 ± 1.44
PK (mmol/L)	4.27 ± 0.12	4.03 ± 0.16	3.99 ± 0.11
PCr (mg/dL)	0.65 ± 0.01	1.53 ± 0.05^*∗∗*^	1.16 ± 0.25
Purea (mg/dL)	20.45 ± 1.53	50.25 ± 5.66^*∗*^	50.77 ± 11.16
UCr (mg/dL)	74.55 ± 6.60	46.55 ± 1.81	101.10 ± 22.05^#^
Uurea (mg/dL)	1368.66 ± 60.54	1196.66 ± 89.98	1873.83 ± 526.57
UNaV° (*μ*mol/min. gKW)	0.36 ± 0.02	0.94 ± 0.17^*∗*^	0.60 ± 0.18
UKV° (*μ*mol/min. gKW)	0.22 ± 0.01	1.30 ± 0.20^*∗∗*^	0.80 ± 0.24

Effect of *SP* extract on some biochemical markers following I/R injury. Data are shown as mean ± SEM. ^*∗*^Significant against the SC group (*P* < 0.05); ^#^significant against the I/R rats (*P* < 0.05). *SP*: *Stachys pilifera*; SC: sham control; I/R: ischemia/reperfusion; KW: kidney weight; UV: urine volume; V°: urine flow rate; PK: plasma K; PNa: plasma Na; Purea: plasma urea; PCr: plasma creatinine; UCr: urine creatinine; Uurea: urine urea; UNaV°: absolute excretion of Na; UKV°: absolute excretion of K.

## Data Availability

The data supporting the finding of this study are available within the article.
